# Lead (Pb) and cadmium (Cd) blood levels and potential hematological health risk among inhabitants of the claimed hazardous region around Qaroun Lake in Egypt

**DOI:** 10.1186/s12889-023-16007-w

**Published:** 2023-06-05

**Authors:** Salwa Bakr, Makram Ahmed Sayed, Karem Mohamed Salem, Enas Mohamed Morsi, Mohamed Masoud, Eman Mahmoud Ezzat

**Affiliations:** 1 Department of Clinical Pathology/ Hematology & Transfusion Medicine, Faculty of Medicine, Fayoum University, P.O Box: 63514, Fayoum, Egypt; 2 Head of Environmental and Food Pollutants Analysis Lab, Faculty of Agriculture, Fayoum University, Fayoum, Egypt; 3 Department of Internal Medicine, Faculty of Medicine, Fayoum University, Fayoum, Egypt; 4 Department of Forensic Medicine and Clinical Toxicology, Faculty of Medicine, Fayoum University, Fayoum, Egypt; 5 Department of Public Health and Community Medicine, Faculty of Medicine, Fayoum University, Fayoum, Egypt

**Keywords:** Lead, Cadmium, Wadi El-Rayan, Qaroun, Egypt

## Abstract

**Background:**

Lead (Pb) and cadmium (Cd) heavy metals are considered potentially hazardous toxins which cause serious health problems. Many studies reported that the water of Qaroun Lake in Fayoum, Egypt with its fish farms was contaminated with Pb and Cd above permissible levels. However, there is a lack of studies addressing levels of these toxic metals among inhabitants.

**Objectives:**

We aimed to evaluate blood levels of Pb and Cd and their potential health risk among inhabitants around Qaroun Lake.

**Materials and methods:**

This case-control study estimated Pb and Cd blood levels among 190 individuals from two destinations (near and far away) of Qaroun Lack using an atomic absorption spectrometer after full history taking and routine checkup investigations; Full blood count, serum ferritin, liver enzyme (ALT), and creatinine levels.

**Results:**

There was a significant difference between blood levels of Pb and Cd heavy metals of inhabitants from near and far away Qaroun Lake destinations (p-value < 0.001). The majority of inhabitants around Qaroun Lake had Pb and Cd blood levels above permissible levels (100% and 60% respectively). Critical levels out of them were 12.1% and 30.3% respectively. In comparison to inhabitants faraway Qaroun Lake, three individuals (2.4%) had Cd above the permissible level, while all of them (100%) had Pb level within the permissible level. There were no statistically significant differences between the two sampled populations as regards hemoglobin level, ALT, creatinine, and ferritin serum levels (p-value > 0.05). The difference between studied populations regarding types of anemia was not statistically significant. Subclinical leucopenia was higher in the population near Qaroun Lake when compare to inhabitants far from the lake (13.6% vs. 4.8%, p-value 0.032).

**Conclusion:**

Bio-monitoring of populations exposed to Pb and Cd hazardous substances could help in generating an early warning system to reduce the disease burden associated with their toxicity.

## Introduction

Cadmium (Cd) and Lead (Pb) heavy metals, which are considered potentially hazardous toxins, cause serious health problems. Lead, cadmium, iron, and zinc were reported to be the commonest toxic metals that affect human health (1). Once a toxic metal enters the body, it distributes through the blood to soft tissues (e.g., liver, kidney, brain), and bones (2). Acute and chronic toxic effects of these heavy elements, as a consequence, affect different body organs causing severe complications such as; gastrointestinal disorders, kidney failure, anemia, vascular damage, birth defects, nervous system disorders, and skin lesions (3, 4).

Pb is primarily toxic to the nervous system but can also induce anemia by disturbing the heme synthesis pathway (5). Cd which has been categorized as carcinogenic for human beings by the International Agency for Research on Cancer is mainly affecting the kidney and can also cause bone demineralization (6).

Pb and Cd heavy metals enter the environment from both industry and natural resources; such as volcanic emissions and weathering of rocks, therefore, they are found in air, soil, and water and can subsequently accumulate in plants and animals (5). In addition, both Pb and Cd have been used in different industries. Although Pb has unique properties in industries, Cd has declined in the United States of America since 2001 in response to environmental pollution concerns. Lead has been used in the manufacturing of batteries, painting, plumbing, mining, pipes, and metal recycling, while Cd is used in nonferrous alloys, batteries, plastic stabilizers, pigment production, coatings, and plating (6).

According to **the Centers for Disease Control and Prevention (CDC)**, the average concentration of blood Pb among healthy unexposed adults was estimated at 0.9 ug/dL. The estimated reference values of permissible Pb blood levels are < 5ug/dL in adults and < 3.5 ug/dL in children, while the critical/ hazardous levels are ≥ 70 ug/dL in adults and ≥ 20 ug/dL in children (7, 8). The average range of Cd blood level of healthy unexposed adults is 0.1–4 µg/L. The estimated reference value of the permissible Cd blood level is ≤ 0.4 ug/L, while the critical value is > 5 ug/dL (9).

Generally, the primary source of Pb and Cd exposure is contaminated food. Leafy vegetables in general including Tobacco leaves contain high levels of Cd due to bioaccumulation from the soil (9, 10, 11). However, in the United States, nonsmoker individuals who regularly consume shellfish and organ meats will have a higher Cd exposure risk (9). Because both Pb and Cd elements have no known biological role in the human body, **the Joint Expert Committee on Food Additives (JECFA)**, an international scientific committee of the Food and Agriculture Organization of the United Nations in collaboration with the World Health Organization has established the safe level of provisional tolerable monthly intake (PTMI) of Cd and Pb heavy elements of 25 µg/kg of body weight (12).

Many studies reported that contamination of freshwater with heavy metals with subsequent contamination of aquatic organisms, particularly fish, has become a matter of critical concern (13, 14). Previous studies that evaluated the level of heavy metal in Lake Qaroun in El-Fayoum Governorate and its fish farms found that some toxic heavy metals, particularly lead, iron, zinc, and cadmium were higher than the permissible level (15, 16, 17, 18). However, there is a lack of studies that evaluated these toxic heavy metals among inhabitants around the tourist Qaroun Lake. Hence, the present study is aiming to evaluate the level of lead and cadmium metals in the blood of inhabitants of the region around Qaroun Lake of Fayoum Governorate in Egypt and to assess their potential health risks.

## Materials and methods

### Study design and sample population

**Fig. 1:**
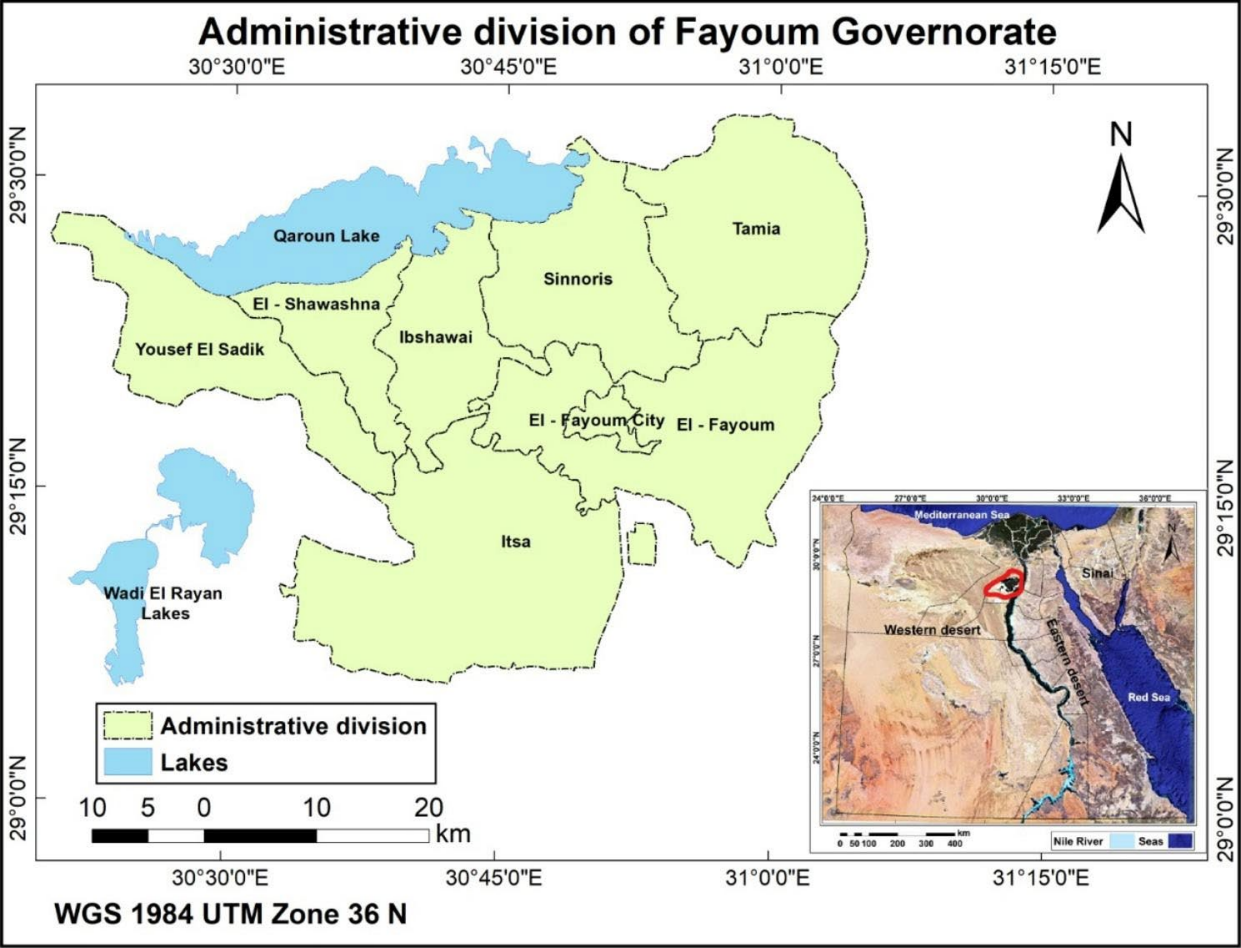
Ibshwai and Tamia Districts located around Qaroun Lake in Fayoum Governorate in Egypt

This case-control study, which was carried out in Fayoum Governorate of Egypt during the period from October to December 2022 from two destinations (near and far away) of Qaroun Lack as per the Declaration of Helsinki ethical standards and after taking the approval of the ethical committee of Faculty of Medicine Fayoum University (Fig. 1). The sample size was calculated using (G power version 3.1.9.4) to get a power level of 0.90, an alpha level of 0.05, and a medium effect size of 0.5 for Pb and Cd blood levels between the two study areas. We assumed 1:2 as a ratio between the two destinations, so the sample size was estimated to be 64 and 128 in the areas near and far away from Qaroun Lake, respectively. The final study sample was 190 participants including 66 and 124 inhabitants from areas near and far away from Qaroun Lake, respectively.

Full history taking and clinical examination that addressed the risk of exposure to Pb and Cd either environmental or occupational risk, as well as, any expected medical outcomes manifestation. Peripheral venous blood samples withdrew from each participant with informed consent into serum and ethylene diamine tetra acetic acid (EDTA) Becton Dickinson vacutainer tubes (BD, Franklin Lakes, NJ, USA). Routine checkup investigations including Full blood count (CBC), serum ferritin, ALT liver enzyme, and serum creatinine levels were performed for the study population. Pb and Cd heavy metals were analyzed in serum samples of the participants using an atomic absorption spectrometer after sample separation and digestion using the ISO standard methods for determination of Pb and Cd levels.

### Metal analysis in blood samples

#### Sample digestion

The sample digestion occurred depending on the ISO standard methods for wet digestion samples DIN EN 13805 (19). A weight in the range of 0.25 g of the sample was placed in the digestion vessel (DAP 60), then 1.25 ml of concentrated nitric acid (Merck, Germany) was added to it. After fumes volatilization under the fume hood, the digestion vessels are sealed and placed in the microwave (Berghof, Germany). The digestion program starts by raising the temperature to 160°C at a rate of 5°C / minute, then increasing to 190°C at a rate of 1°C/minute. At the end of the digestion cycle, the temperature is reduced to 75°C at the rate of 1°C/minute.

#### Determination of lead and cadmium concentration

Agilent atomic absorption spectrometer equipped by graphite furnace unit model 240ZAA (Agilent Technologies, Germany) was used to determine cadmium (Cd) and lead (Pb) concentrations according to EN 14084:2003 (20). The temperature program used for these elements is as fellow:

**Table:** 

step	Cadmium (Cd)			Lead (Pb)		
	Temp. (°C)	Time (sec)	Flow (L/min)	Temp. (°C)	Time (sec)	Flow (L/min)
1	85	5	0.3	85	5	0.3
2	95	40	0.3	95	40	0.3
3	120	10	0.3	120	10	0.3
4	250	5	0.3	400	5	0.3
5	250	1	0.3	400	1	0.3
6	250	2	0	4000	2	0
7	1800	0.8	0	2100	0.9	0
8	1800	2	0	2100	2	0
9	1800	2	0.3	2100	2	0.3

The used method was validated using the average percent recovery and standard deviation plotted into a quality control chart. The quality control sample was carried out with every set of samples by spiking the sample with known concentrations of lead and cadmium. The data of the quality control samples was used to calculate recovery percent, which was determined as an average of 95% and to ensure the accuracy and the precision of the method used.

#### Statistical data analysis

Statistical Package for Social Sciences (SPSS 22) was used for data statistical analysis. For lead and cadmium levels, median and interquartile range (IQR) were estimated. In comparing the two studied inhabitants, the Mann-Whitney U test was used. Categorical data were presented as frequencies and percentages. For comparing categorical data, the χ2 test or Fischer exact test was performed. P values less than 0.05 was considered to be statistically significant.

## Results

The present study enrolled 190 residents aged (3–80 years) from two different districts located around Qaroun Lake in Fayoum Governorate in Egypt, where Ibshwai province located nearby the lake and Tamia province located far away from the lake were selected (Fig. 1). Only (10%) of the total population practiced risky occupations relevant to Pb and Cd heavy metal toxicity. There were no significant differences between the two studied populations as regards age, sex, house, and occupation. There were statistically significant differences between the two studied groups as regards working in farming, smoking, and consumption of fish being higher in inhabitants of regions far from Qaroun Lake (p-value: 0.037, 0.001, 0.029 respectively) (Table 1). The prevalence of abortion and birth defects was higher in areas far from the lake as compared to those near the lake. The sociodemographic and clinical characteristics of the two groups of the study population are presented in Table (1).

**Table 1:** Sociodemographic and Clinical Characteristics among Sample Population:

Variables		Population Near Qaroun Lake (n = 66)	Population Distant Qaroun Lake (n = 124)	P-value
		No. (%)	No. (%)	
**Age (years)**	3–17	15 (22.7%)	23 (18.5%)	0.546
	18–39	27 (40.9%)	50 (40.3%)	
	40–59	23 (34.8%)	44 (35.5%)	
	60–80	1 (1.5%)	7 (5.6%)	
**Sex**	Male	21 (31.8%)	46 (37.1%)	0.468
	Female	45 (68.2%)	78 (62.9%)	
**Occupation**	Not working	56 (84.8%)	85 (68.5%)	0.051
	Risky**	4 (6.1%)	15 (12.1%)	
	Not risky	6 (9.1%)	24 (19.4%)	
**Previous working in farming**	Yes	1 (1.5%)	13 (10.5%)	0.037*
	No	65 (98.5%)	111 (89.5%)	
**Previous work in painting**	yes	0 (0%)	1 (0.8%)	1.000
	No	66 (100%)	123 (99.2%)	
**Smoking**	Smoker	0 (0%)	18 (14.5%)	0.001*
	Non-smoker	65 (98.5%)	98 (79.0%)	
	Ex-smoker	1 (1.5%)	8 (6.5%)	
**House**	Old	33 (50%)	72 (58.1%)	0.287
	New	33 (50%)	52 (41.9%)	
**Eating fish**	Once/ week	5 (7.6%)	11 (8.9%)	0.029*
	3 times/ month	7 (10.6%)	25 (20.2%)	
	2 times /month	33 (50%)	70 (56.5%)	
	Never	21 (31.8%)	18 (14.5%)	
**Abortion**	Yes	6 (13.3%)	25 (32.1%)	0.021*
	No	39 (86.7%)	53 (67.9%)	
**Birth defects**	Yes	2 (4.4%)	23 (29.5%)	0.001*
	No	43 (95.6%)	55 (70.5%)	
**Chronic diseases**	Diseased	8 (12.1%)	19 (15.3%)	0.547
	Non-diseased	58 (87.9%)	105 (84.7%)	
**HTN**	Hypertensive	1 (1.5%)	14 (11.3%)	0.017*
	Non-hypertensive	65 (98.5%)	110 (88.7%)	
**DM**	Diabetic	1 (1.5%)	7 (5.6%)	0.177
	Non-diabetic	65 (98.5%)	117 (94.4%)	

*Significant at p < 0.050, ** Occupations related to exposure to heavy metals, ***IDA: Iron deficiency anemia

Although more than one-third of the study population (74/190, 38.9%) was anemic, only less than one-fifth of them (14.3%) had chronic diseases with no statistically significant differences between the two studied populations (p-value 0.594 and 0.547 respectively) (Table 1).

Regarding hematological lab findings, out of a total of 66 inhabitants with microcytic hypochromic anemia, only 24 (12.63%) were iron deficiency anemia (IDA) confirmed with serum ferritin levels below normal cutoff value for gender and age. Additionally, subclinical leucopenia was higher in the population near Qaroun Lake when compare to those far from the lake (13.6% vs. 4.8% with a p-value of 0.032) (Table 2). Regarding liver and renal function tests, there were no statistically significant differences between the two sampled populations as regards ALT, creatinine, and ferritin (p-value > 0.05) (Table 2).

**Table 2:** Comparison between Hematological and Routine Laboratory Checkup Parameters of Sample Population:

Laboratory Investigations	Population Around Qaroun Lake (n = 66)	Population Distant Qaroun Lake (n = 124)	P- value
	No. (%)	No. (%)	
**White Blood Cell Count** - Normal count (4–11 10^9^/L) - Leucopenia	57 (86.4%)	118 (95.2%)	0.032****
	9 (13.6%)	6 (4.8%)	
**Hemoglobin Conc.** - Normal conc. (11.5–17.5 g/dL) - Anemic level	42 (63.6%)	74 (59.7%)	0.594
	24 (36.4%)	50 (40.3%)	
Subclinical MHA*: **-** Anemic - Non-anemic	23 (34.8%)	43 (34.7%)	0.981
	43 (65.2%)	81 (65.3%)	
Subclinical IDA** - Iron deficiency - Non-iron deficiency	5 (21.7%)	19 (44.2%)	0.071
	18 (78.3%)	24 (55.8%)	
Mentzer screening index (< 14) *** - Positive - Negative	1 (4.4%)	7 (16.3%)	0.179
	22 (95.6%)	36 (83.7%)	
**MHA with uncertain etiology#**	17 (73.9%)	17 (39.5%)	0.039****
**Serum Ferritin** - Normal range (Male: up to 300 – Female: up to 200 ug/L) - Low level - High level	49 (74.2%)	90 (72.6%)	0.970
	16 (24.2%)	32 (25.8%)	
	1 (1.5%)	2 (1.6%)	
**ALT** - Normal range (Up to 40 IU/L) - High serum level	62 (93.9%)	118 (95.2%)	0.719
	4 (6.1%)	6 (4.8%)	
**Serum Creatinine** - Normal range (Male: 1.1–1.4 mg/dl; Female: 0.6–1.1 mg/dl) - High serum level	65 (98.5%)	116 (93.5%)	0.127
	1 (1.5%)	8 (6.5%)	

*MHA: Microcytic Hypochromic Anemia among the anemic study population. **IDA: Iron deficiency anemia. ***Mentzer index (MCV/ RBCs) calculated among individuals with microcytic hypochromic anemia < 14 suggestive of thalassemia trait. ****Significant at p < 0.050. # MHA after exclusion of those cases due to IDA and positive Mentzer with suspicious thalassemia trait

**Fig. 2:**
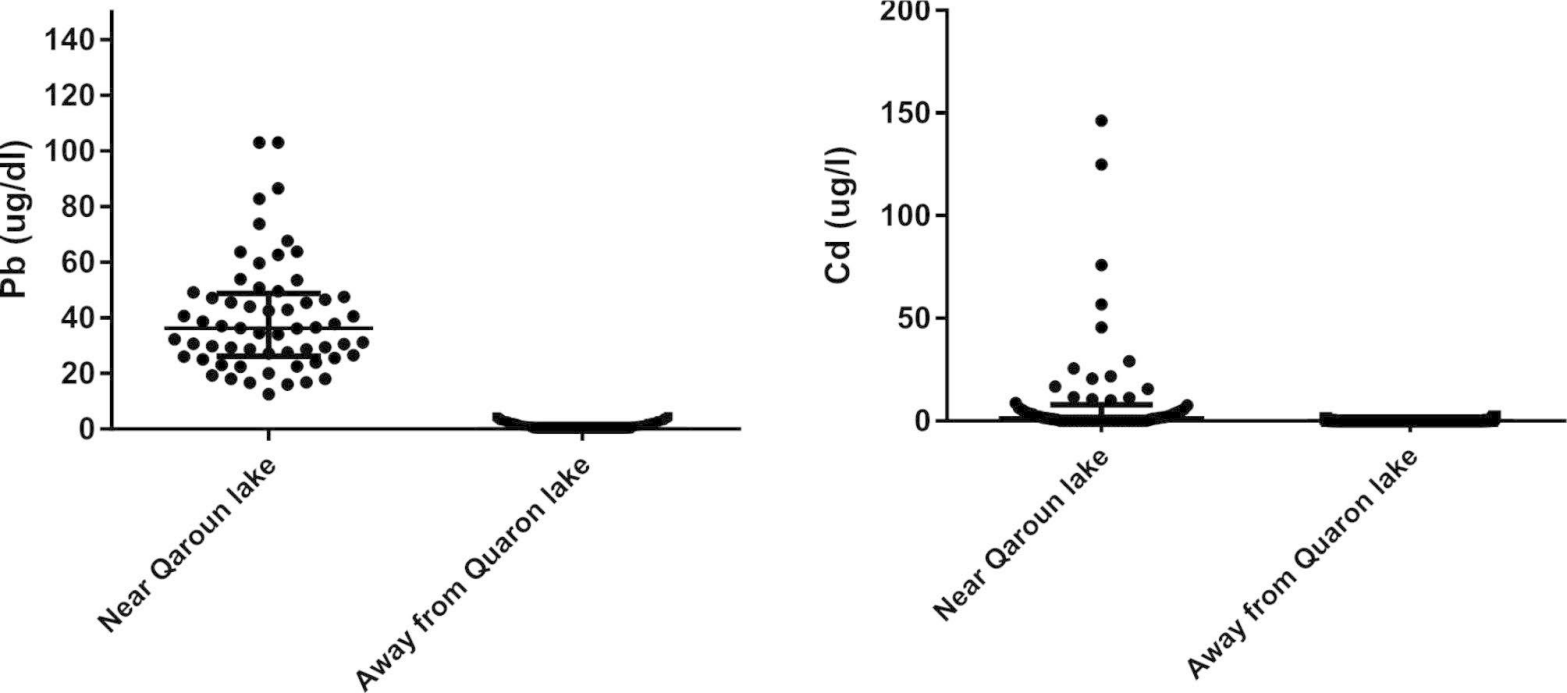
Jitter plot with median (IQR) for blood Pb and Cd levels in both study population

There was a significant difference between blood levels of Pb and Cd elements of inhabitants from near and far away Quaron Lake destinations (p-value < 0.001). The majority of inhabitants around Qaroun Lake had Pb and Cd blood levels much above permissible levels established by the CDC (100% and 60.6% respectively). Critical levels out of them were 12.1% and 30.3% respectively. In comparison to inhabitants faraway Qaroun Lake, three individuals (2.4%) had Cd above the permissible level, while all of them (100%) had Pb level within the permissible level (Table 3). The median (IQR) blood concentration for Pb and Cd among our study population near Qaroun Lake was 36.25 (26.20–48.78) ug/dL and 1.45 (0–7.90) ug/dL, respectively. Whereas, their median (IQR) values among those who live far away from the lake were 0 (0–1.7) ug/L and 0 (0–0) ug/L, respectively (Fig. 2).

**Table 3:** Prevalence of Pb and Cd in Blood of Sample Population

Blood/ Serum Level		Population Around Qaroun Lake (n = 66)	Population Distant Qaroun Lake (n = 124)	P- value
		No. (%)	No. (%)	
**Lead** (**Pb**)	Permissible blood level (Children < 3.5 ug/dl; Adult < 5 ug/dl)	0 (0%)	124 (100%)	< 0.001*
	Above permissible level	58 (87.9%)	0 (0%)	
	Critical/ Hazardous level (Children ≥ 20 ug/dl; Adult ≥ 70 ug/dl)	8 (12.1%)	0 (0%)	
**Cadmium (Cd)**	Permissible blood level (≤ 0.4 ug/L)	26 (39.4%)	121 (97.6%)	< 0.001*
	Above permissible level	20 (30.3%)	3 (2.4%)	
	Critical/ Hazardous level (≥ 5 ug/L)	20 (30.3%)	0 (0%)	

*Significant at p < 0.050

In inhabitants around Qaroun Lake, our results found that the critical Pb levels were detected more among younger age groups rather than other age categories (p-value 0.036) (Table 4), however, there were no statistically significant differences between the studied heavy metals (Pb and Cd) blood levels and occurrence of noteworthy clinical/ subclinical manifestations (p-value > 0.05) (Table 5). Though the most obvious clinical/ subclinical manifestation among them was microcytic hypochromic anemia, the majority (17 out of 66 individuals, 73.91%) of them were diagnosed without a clear conclusive underlying etiology (Table 2). Out of a total of 23 inhabitants with microcytic anemia, only 5 (21.7%) had iron deficiency and another had suspicious thalassemia traits by using the Mentzer screening index [21].

**Table 4:** Association between Impermissible Blood Levels of Pb and Cd and Basic Characteristics of Inhabitant near Qaroun Lake in Fayoum Governorate:

Variables		Lead (Pb) (n = 66)		Cadmium (Cd) (n = 66)	
		**Critical** (n = 8)	**Under-critical/ Impermissible** (n = 58)	**Critical/ Impermissible** (n = 40)	**Permissible** (n = 26)
		**No. (%)**	**No. (%)**	**No. (%)**	**No. (%)**
**Age (years)**	3–17	5 (62.5%)	10 (17.2%)	10 (25.0%)	5 (19.2%)
	18–39	1 (12.5%)	26 (44.8%)	15 (37.5%)	12 (46.2%)
	40–59	2 (25.0%)	21 (36.2%)	15 (37.5%)	8 (30.8%)
	60–80	0 (0.0%)	1 (1.7%)	0 (0.0%)	1 (3.8%)
**P-value**		0.036*		0.520	
**Sex**	Male	4 (50.0%)	17 (29.3%)	13 (32.5%)	8 (30.8%)
	Female	4 (50.0%)	41 (70.7%)	27 (67.5%)	18 (69.2%)
**P-value**		0.253		0.883	
**Occupation**	Not working	8 (100%)	48 (82.8%	33 (82.5%)	23 (88.5%)
	Risky occupation	0 (0.0%)	4 (6.9%)	3 (7.5%)	1 (3.8%)
	Non-Risky occupation	0 (0.0%)	6 (10.3%)	4 (10.0%)	2 (7.7%)
**P-value**		0.444		0.777	
**House**	Old	3 (37.5%)	30 (51.7%)	21 (52.5%)	12 (46.2%)
	New	5 (62.5%)	28 (48.3%)	19 (47.5%)	14 (53.8%)
**P-value**		0.708		0.614	
**Eating fish**	One/week	0 (0.0%)	5 (8.6%)	1 (2.5%)	4 (15.4%)
	Three/month	1 (12.5%)	6 (10.3%)	2 (5.0%)	5 (19.2%)
	Two/month	5 (62.5%)	28 (48.3%)	24 (60.0%)	9 (34.6%)
	Never	2 (25.0%)	19 (32.8%)	13 (32.5%)	8 (30.8%)
**P-value**		0.768		0.037*	
**Abortion**	Yes	0 (0.0%)	6 (15.0%)	4 (15.4%)	2 (11.1%)
	No	4 (100.0%)	34 (85.0%)	22 (84.6%)	16 (88.9%)
**P-value**		1.000		1.000	
**Birth defects**	yes	0 (0.0%)	2 (5.0%)	1 (3.8%)	1 (5.6%)
	No	4 (100.0%)	38 (95.0%)	25 (96.2%)	17 (94.4%)
**P-value**		1.000		1.000	

*Significant at p < 0.050

**Table 5:** Association between Pb and Cd Blood Levels and Clinical/ Subclinical Manifestation of Inhabitants near Qaroun Lake in Fayoum Governorate:

Clinical/ Subclinical Manifestation		Lead (Pb) (n = 66)		Cadmium (Cd) (n = 66)	
		**Critical** (n = 8)	Under-critical/ Impermissible* (n = 58)	**Critical/ Impermissible** (n = 40)	**Permissible** (n = 26)
		**No. (%)**	**No. (%)**	**No. (%)**	**No. (%)**
**History of any Chronic diseases**	Yes	1 (12.5%)	7 (12.1%)	3 (7.5%)	5 (19.2%)
	No	7 (87.5%)	51 (87.9%)	37 (92.5%)	21 (80.8%)
**P-value**		1.000		0.247	
**History of HTN**	Yes	0 (0.0%)	1 (1.7%)	1 (2.5%)	0 (0.0%)
	No	8 (100.0%)	57 (98.3%)	39 (97.5%)	26 (100.0%)
**P-value**		1.000		1.000	
**History of DM**	Yes	0 (0.0%)	1 (1.7%)	1 (2.5%)	0 (0.0%)
	No	8 (100.0%)	57 (98.3%)	39 (97.5%)	26 (100.0%)
**P-value**		1.000		1.000	
**Microcytic Hypochromic Anemia**	Present	3 (37.5%)	20 (36.2%)	12 (30.0%)	11 (42.3%)
	Absent	5 (62.5%)	38 (63.8%)	28 (70.0%)	15 (57.7%)
**P-value**		1.000		0.305	
**Subclinical IDA**	Present	1 (33.3%)	4 (20.0%)	1 (8.3%)	4 (36.4%)
	Absent	2 (66.7%)	16 (80.0%)	11 (91.7%)	7 (63.6%)
**P-value**		0.539		0.155	
**Subclinical Leucopenia**	Present	0 (0.0%)	9 (15.5%)	7 (17.5%)	2 (7.7%)
	Absent	8 (100.0%)	49 (84.5%)	33 (82.5%)	24 (92.3%)
**P-value**		0.586		0.465	
**ALT Liver Enzyme**	High	0 (0.0%)	4 (6.9%)	3 (7.5%)	1 (3.8%)
	Normal	8 (100.0%)	54 (93.1%)	37 (92.5%)	25 (96.2%)
**P-value**		1.000		1.000	
**Serum Creatinine**	High	0 (0.0%)	1 (1.7%)	0 (0.0%)	1 (3.8%)
	Normal	8 (100.0%)	57 (98.3%)	40 (100.0%)	25 (96.2%)
**P-value**		1.000		0.394	
**Serum Ferritin**	Normal	4 (50.0%)	45 (77.6%)	30 (75.0%)	19 (38.8%)
	Low	4 (50.0%)	12 (20.7%)	9 (22.5%)	7 (26.9%)
	High	0 (0.0%)	1 (1.7%)	1 (2.5%)	0 (0.0%)
**P-value**		0.187		0.675	

*None of the inhabitants had lead levels within the permissible level

Regarding those inhabitants having microcytic hypochromic anemia with uncertain etiology, there was a statistically significant difference between them being higher among those living near Qaroun Lake with a p-value of 0.039 (Table 2). Out of those 17 individuals with microcytic hypochromic anemia with inconclusive etiology lived near the lake, 17 (100%) had Pb and 5 (29.41%) had Cd above the permissible level.

## Discussion

There is no data in the literature on the potential health risks of Pb and Cd heavy metals in the Egyptian population particularly in the region claimed to be potentially polluted, around Qaroun Lake in Egypt. Nevertheless, the previously published studies on the pollution of Lake Qaroun with heavy metals particularly Pb and Cd have been noted [15–18, 22]. Hence, in this study, we aimed to evaluate the most hazardous of these claimed elements (Pb and Cd) in the blood of 190 inhabitants in Fayoum Governorate from different regions using an atomic absorption spectrometer after sample digestion. This study will be considered a preliminary study to assess the commonest hazardous heavy metals of Lack Qaroun of Fayoum Governorates in Egypt.

The historically renowned Qaroun Lake, a close ecosystem in nature, is located in the north of Fayoum Governorate in Egypt (Fig. 1), where contaminants from El-Bats and El-Wadi drains discharge along with agriculture drainage water of Fayoum province [17]. It was reported that such anthropogenic inputs and/ or geological sources contribute to the biodiversity of the water lake, where its original freshwater turned into the currently brackish one. Additionally, the evaporation of water due to the hot climate of the region augments the intensification of heavy metals, salts, and pesticide concentrations in the lake [15]. Accordingly, such heavy metals contaminations in Qaroun Lake may have devastating effects on the ecological balance of the aquatic environment, hence on the environmental media such as dust, air, soil, and others [14, 23].

Our study results coincide with previous studies in which Pb and Cd concentrations in water samples collected from Wadi EL-Rayan Lakes were higher than the maximum permissible level recommended by Egyptian Environmental law, (48) decision 1992/2013, in the spring/ hot season, but within permissible levels in cold one [15, 16, 22]. Furthermore, in correlation, they found that urea and creatinine serum levels in Tilapia fish showed a significant increase. On the other hand, **George and his colleagues (2013)** reported that Cd concentration in the water samples collected from Wadi El-Rayan Lakes was within the recommended permissible limits (100 µg/L) of the National Environmental Law [24], where it ranged from (12 to 16.8 µg/L) in the cold season and from (15.7 to 30.7 µg/L) in the hot one [16].

In our study, the median (IQR) blood concentration for Pb among our study population lived near Qaroun Lake was 36.25 (26.20–48.78) ug/dL, while it was 0 (0–1.7) ug/dL among those who lived far away the lake. Although various studies disclosed that the normal value for blood Pb concentration was 42.6 µg/L, different values were also reported for other countries. The mean Pb level in an Amazonian district was 32.77 µg/L, while it was 14.5 µg/L in Australia, 21.3 µg/L in Canada, 12.3 µg/L in the Americas, 65.4 µg/L in Brazil, 33.4 µg/L in Italy, and 19.1 µg/L in Korea [11]. In conclusion, our results showed in agreement with previous studies that the level of blood Pb concentration might be influenced by numerous epidemiological and environmental variables such as diet, age, and gender [25]. Regarding the median (IQR) of Cd blood level, the value was 1.45 (0–7.90) µg/L among those who lived nearby the lake, but an undetectable level was noted among those who lived far away from the claimed destination of the Qaroun Lake region.

Furthermore, our results found that there were no significant differences between the population who lived nearby Qaroun Lake compared to those who lived far away the lack as regards age, gender, house, and occupation. However, we found that there was a significant difference between blood levels of Pb and Cd heavy elements of inhabitants from the two destinations around Quaron Lake (p-value < 0.001). Inconsistent with previous studies described from Bangladesh (26), Mexico (27), and Russia (28), our results showed that the majority of inhabitants nearby Quaron Lake who had Pb and Cd blood levels much above permissible levels established by the CDC were among the younger age group (p-value 0.036). Although the developing biological systems of the human body particularly the nervous system in children is most sensitive to the effects of Pb blood level (9), previous studies similarly revealed that the mean blood lead level was higher in children aged from 6 to 11 years than other age categories (29). There is no acknowledged safe blood Pb concentration in children, where cognitive, behavioral, and learning difficulties were reported with low blood Pb concentration even as low as 5ug/dL, the CDC (2021) updated the blood lead reference value in children to 3.5 µg/dL instead of 5 µg/dL for avoiding the serious consequence of lead exposure on children’s health (7).

On the contrary, **Queiroz and his colleagues (2019)** reported that blood Pb levels show trends with age and gender being higher in adults and the elderly (11, 30–32). This point out that the duration of exposure to lead in a residential area is consistently found to be a strong predictor of high blood Pb level (33–35). Regarding gender variant in this study, though the mean blood Pb and Cd critical levels were not statistically different between males and females, the prevalence was almost the same regarding the critical level of blood Pb level being equal (50%) in both gender but and the prevalence of elevated blood Cd level in females was higher than males. This might be explained by the higher number of females who participated in this study in comparison to males. Controversial results were noted in previous studies conducted in Egypt (29, 36), Bangladesh (37), Thailand (38), and Pakistan (33), where the incidence of elevated blood lead concentration was higher in males than females. This might be explained by the different traditional roles of male and female in these societies, where increased outdoor activities and occupational risk for males appears to be involved in this sex-related difference (29).

Independent of the level of lead heavy metal intoxication in human body fluids, it was reported that the appearance of clinical manifestations varies from one individual to another depending on the surrounding environmental differences [31]. Though the most obvious clinical/ subclinical manifestation among inhabitants living near Qaroun Lake was microcytic hypochromic anemia, the majority (73.91%) of them were diagnosed without a clear conclusive underlying etiology. Regarding those inhabitants having microcytic hypochromic anemia with uncertain etiology, there was a statistically significant difference between them being higher among those living near Qaroun Lake with a p-value of 0.039. Out of those 17 individuals with microcytic hypochromic anemia with inconclusive etiology lived near the lake, had 17 (100%) Pb and 5 (29.41%) Cd above the permissible levels. Additionally, subclinical leucopenia was noted higher in inhabitants of the region nearby Qaroun Lake compared to those far away from the lake (13.6% vs. 4.8% with a p-value of 0.032). Similarly, several published studies have noted that cumulative Pb poisoning is associated with increased potential health risks for hematological disorders particularly anemia [36, 39, 40]. However, others reported a non-significant association between the presence of anemia and elevated blood Pb level [36, 41].

Though there is no safe level of Pb blood level, previous studies have shown that lead-induced anemia microcytic hypochromic can be easily diagnosed at Pb blood levels higher than 50 µg/dL in adults [42]. It was reported that anemia-associated chronic exposure to Pb is the result of both interfering with heme biosynthesis and shortening of red blood cell lifespan [43]. The critical effect of Pb in the human body is mainly attributed to cellular enzymatic disruption of δ-aminolevulinic acid dehydratase (ALAD) that is essential in heme biosynthesis with a consequent decrease in various red blood cell hematological parameters (RDW-CV, MCV, MCH, and hematocrit) and potential risk of anemia [9, 44, 45].

For the well-known Pb and Cd biogeochemical cycle, these heavy metals are usually absorbed from regional background levels and might be present in drinking water, household dust, and food in which environmental media such as dust, air, soil, and others may have an excess of these toxic heavy elements. According to a study carried out in the region around Qaroun Lake in Fayoum Governorate a region with a characteristic ecosystem, geology, and residency might contribute to overt circulating Pb and Cd levels in the region [11]. This might explain the statistically significant differences between the two studied groups as regards working in farming, consumption of fish, smoking, and hypertension despite being higher in inhabitants of the region far from Qaroun Lake.

Though there were no statistically significant difference significant differences between the studied heavy metals (Pb and Cd) blood levels and occurrence of noteworthy clinical/ subclinical manifestations in both kidney and liver functions when ALT, creatinine, and ferritin serum levels have been estimated in all the study population (p-value > 0.05). The present study results, in agreement with previous studies, are considered an alarm to the toxic effect of environmental changes in Qaroun Lack ecosystem for being the final reservoir for most of such heavy metals, where it receives heavy loads of organic and inorganic pollutants via several agricultural drains and huge amounts of raw sewage, agricultural and industrial wastewater discharged into the lake [46, 47].

Bio-monitoring of populations exposed to Pb and Cd hazardous substances could help in generating an early warning system to reduce the disease burden associated with their toxicity. However, a mass screening program for both inhabitants and environmental media of the regions nearby Qaroun Lake and Wadi El-Rayan in Fayoum Governorate is mandatory to validate such heavy metals’ hazardous impact.

## Conclusion

This study could be a preliminary study for bio-monitoring of the Fayoum population who are exposed to the hazardous effect of heavy metals in Lake Qarun water and its farm fish. Hence, to generate an early warning system to reduce the disease burden associated with their increased level and lethal toxicity; such as Alzheimer, kidney failure, and anemia for unknown causes, that are otherwise easily preventable.

## Data Availability

All relevant data are within the paper.

## References

[1] Luo L, Wang B, Jiang J, Fitzgerald M, Huang Q, Yu Z, Li H, Zhang J, Wei J, Yang C, Zhang H. Heavy metal contaminations in herbal medicines: determination, comprehensive risk assessments, and solutions. Front Pharmacol 2021 Jan 14;11:595335. 10.3389/fphar.2020.595335 PMC7883644 33597875

[2] El-Sawi I, El-Saied M (2013). Umbilical cord-blood lead levels and pregnancy outcome. J Pharmacol Toxicol.

[3] Gazwi HSS, Yassien EE, Hassan HM. Mitigation of lead neurotoxicity by the ethanolic extract of Laurus leaf in rats. Ecotoxicol Environ Saf. 2020 Apr;1192:110297. 10.1016/j.ecoenv.2020.110297 32061979

[4] Tsai MT, Huang SY, Cheng SY. Lead poisoning can be easily misdiagnosed as acute porphyria and nonspecific abdominal painCase reports in emergency medicine 2017. Case Rep. Emerg Med. 2017 May 29, 2017. 10.1155/2017/9050713 PMC5467293 28630774

[5] International Agency for Research on Cancer (1993). Beryllium, cadmium, mercury, and exposures in the glass. Apresentado em: IARC Working Group on the evaluation of carcinogenic risks to humans.

[6] Briffa J, Sinagra E, Blundell R. Heavy metal pollution in the environment and their toxicological effects on humans. Heliyon. 2020 Sep 1;6(9):e04691. 10.1016/j.heliyon.2020.e04691 PMC7490536 32964150

[7] The Center for Disease Control and Prevention (CDC). Childhood lead poisoning prevention, blood lead references value. :text=CDC%20uses%20a%20blood%20lead,higher%20than%20most%20children’s%20levels; 2022. https://www.cdc.gov/nceh/lead/data/blood-lead-reference-value.htm#:~

[8] The Center for Disease Control and Prevention (CDC). National Institute of Occupational Safety and Health (NIOSH). Adult Blood Lead Epidemiology and Surveillance (ABLES)., 2022. https://www.cdc.gov/niosh/topics/ables/related.html

[9] The Center for Disease Control and Prevention (CDC). Blood metals panels in the whole blood 2011–2012. https://www.cdc.gov/nchs/data/nhanes/nhanes_11_12/PbCd_G_met_blood%20metals.pdf

[10] Shen X, Chi Y, Xiong K. The effect of heavy metal contamination on humans and animals in the vicinity of a zinc smelting facility. PLoS ONE. 2019 Oct;28(10):e0207423. 10.1371/journal.pone.0207423 PMC6816550 31658263

[11] Queiroz TK, Naka KS, Mendes LD, Costa BN, Jesus IM, Câmara VD, Lima MD. Human blood lead levels and the first evidence of environmental exposure to industrial pollutants in the Amazon. Int J Environ Res Public Health. 2019 Sep;16(17):3047. 10.3390/ijerph16173047 PMC6747206 31443420

[12] Joint FA, World Health Organization, WHO Expert Committee on Food Additives. Evaluation of certain food additives and contaminants: seventy-third [73rd] report of the Joint FAO/WHO Expert Committee on Food additives. World Health Organization; 2011.

[13] Alibabić V, Vahčić N, Bajramović M. Bioaccumulation of metals in fish of Salmonidae family and the impact on fish meat quality. Environ Monit Assess. 2007 Aug;131:349–64. 10.1007/s10661-006-9480-6 17171264

[14] Gad NS (2010). Organochlorine pesticides and trace metals contamination in some marketable fish in Egypt. Egypt J Aquat Res.

[15] Ali M, Abdel-Satar A (2005). Studies of some heavy metals in water, sediment, fish and fish diets in some fish farms in El-Fayoum province. Egypt J Aquat Res.

[16] Mohamed FK, Gad NS, Atrees SS, Hassan NS. Assessment of some Heavy Metals in Water and Tissues of Tilapia Spp. Collected from Wadi El-Rayan Lakes. El-Fayoum Egypt and their Impacts on some Biochemical Parameters. Egyptian Academic Journal of Biological Sciences. C, Physiology and Molecular Biology. 2018 Dec 1;10(2):63–81.

[17] Redwan M, Elhaddad E. Heavy metals seasonal variability and distribution in Lake Qaroun sediments, El-Fayoum, Egypt. J Afr Earth Sc. 2017 Oct;1:134:48–55.

[18] Abdou KH, Moselhy AR, Manal W, Ehdaa MM. Estimation of some heavy metals’ concentration in layer farms at El-Fayoum governorate. J Veterinary Med Res. 2018 Dec;25(1):238–48.

[19] DIN EN 13805 (2014). Foodstuffs—determination of Trace Elements—Pressure digestion, german version DIN EN 13805:2014-12.

[20] European Committee for standardization. Foodstuffs—determination of trace elements—determination of lead, cadmium, zinc, copper and iron by atomic absorption spectrometry (AAS) after microwave digestion. (EN 14084. :2003). (2003).

[21] Sundh A, Kaur P, Palta A, Kaur G. Utility of screening tools to differentiate beta thalassemia trait and iron-deficiency anemia-do they serve a purpose in blood donors. Blood research. 2020 Sep 30;55(3):169 – 74. 10.5045/br.2020.2020219 PMC7536563 32989178

[22] Mohamedien L, Medani G, Gamal Eldien M, Hammad M. Assesment of some Heavy Metals in Water, Sediments and Fish during 2013 in Lake Quaron Protected Area, Fayoum, Egypt. Suez Canal Veterinary Medical Journal. SCVMJ. 2015 Dec;31(2):155–74.

[23] Karak T, Bhattacharyya P. Heavy metal accumulation in soil amended with roadside pond sediment and uptake by winter wheat (Triticum aestivum L. cv. PBW 343). Sci World J. 2010 Jan;1:10:2314–29. 10.1100/tsw.2010.220 PMC5763997 21170482

[24] George U, Asuquo F, Idung J, Andem A (2013). Bioaccumulation of heavy metals in three fresh water fishes caught from Cross River system. Eur J Exp Biol.

[25] Almerud P, Zamaratskaia G, Lindroos AK, Bjermo H, Andersson EM, Lundh T, Ankarberg EH, Lignell S. Cadmium, total mercury, and lead in blood and associations with diet, sociodemographic factors, and smoking in swedish adolescents. Environ Res. 2021 Jun;1:197:110991. 10.1016/j.envres.2021.110991 33705767

[26] Kaiser R, Henderson AK, Daley WR (2001). Blood lead levels of primary school children in Dhaka, Bangladesh. Environ Health Perspect.

[27] Romieu I, Palazuelos E, Meneses F (1992). Vehicular traffic as a determinant of blood lead levels in children: a pilot study in Mexico City. Arch Environ Health.

[28] Rubin CH, Esteban E, Jones R (1997). Childhood lead poisoning in Russia: a site-specific pediatric blood lead evaluation. Int J Occup Environ Health.

[29] Moawad EM, Badawy NM, Manawill M. Environmental and occupational lead exposure among children in Cairo, Egypt: a community-based cross-sectional study. Medicine 2016 Mar;95(9). 10.1097/MD.0000000000002976 PMC4782899 26945415

[30] Rahimpoor R, Rostami M, Assari MJ, Mirzaei A, Zare MR (2020). Evaluation of blood lead levels and their effects on hematological parameters and renal function in iranian lead mine workers. Health Scope.

[31] Wani AL, Ara A, Usmani JA (2015). Lead toxicity: a review. Interdiscip Toxicol.

[32] Jaishankar M, Tseten T, Anbalagan N, Mathew BB, Beeregowda KN. Toxicity, mechanism and health effects of some heavy metals. Interdisciplinary Toxicol. 2014 Jun;7(2):60. 10.2478/intox-2014-0009 PMC4427717 26109881

[33] Khan DA, Qayyum S, Saleem S, Ansari WM, Khan FA (2010). Lead exposure and its adverse health effects among occupational worker’s children. Toxicol Ind Health.

[34] Sharaf NE, Abdel-Shakour A, Amer NM, Abou-Donia MA, Khatab N. Evaluation of children’s blood lead level in Cairo, Egypt. American-Eurasian J Agric Environ Sci. 2008 Jan 1;3(3):414-9.

[35] Paoliello MM, De Capitani EM, Da Cunha FG, Matsuo T, de Fátima Carvalho M, Sakuma A, Figueiredo BR. Exposure of children to lead and cadmium from a mining area of Brazil. Environmental Research. 2002 Feb 1;88(2):120-8. 10.1006/enrs.2001.4311 11908937

[36] Hegazy AA, Zaher MM, Abd El-Hafez MA, Morsy AA, Saleh RA. Relation between anemia and blood levels of lead, copper, zinc and iron among children. BMC Res Notes. 2010 Dec;3:1–9. 10.1186/1756-0500-3-133 PMC2887903 20459857

[37] Mitra AK, Haque A, Islam M, Bashar SA. Lead poisoning: an alarming public health problem in Bangladesh. Int J Environ Res Public Health. 2009 Jan;6(1):84–95. 10.3390/ijerph6010084 PMC2672336 19440271

[38] Neesanan N, Kasemsup R, Ratanachuaeg S, Kojaranjit P, Sakulnoom K, Padungtod C. Preliminary study on assessment of lead exposure in thai children aged between 3–7 years old who live in Umphang district, Tak Province. J Med Assoc Thai. 2011 Aug;94(1):113–20. 22043763

[39] Jain NB, Laden F, Guller U, Shankar A, Kazani S, Garshick E. Relation between blood lead levels and childhood anemia in India. Am J Epidemiol. 2005 May;15(10):968–73. 10.1093/aje/kwi126 15870161

[40] Schwartz J, Landrigan PJ, Baker EL Jr, Orenstein WA, Von Lindern IH. Lead-induced anemia: dose-response relationships and evidence for a threshold. Am J Public Health. 1990 Feb;80(2):165–8. 10.2105/ajph.80.2.165 PMC1404621 2297059

[41] Froom P, Kristal-Boneh E, Benbassat J, Ashkanazi R, Ribak J. Lead exposure in battery-factory workers is not associated with anemia. J Occup Environ Med. 1999 Feb;1:120–3. 10.1097/00043764-199902000-00007 10029957

[42] Rahimpoor R, Rostami M, Assari MJ, Mirzaei A, Zare MR (2020). Evaluation of blood lead levels and their effects on hematological parameters and renal function in iranian lead mine workers. Health Scope.

[43] Hsieh NH, Chung SH, Chen SC, Chen WY, Cheng YH, Lin YJ, You SH, Liao CM. Anemia risk in relation to lead exposure in lead-related manufacturing. BMC Public Health. 2017 Dec;17:1–2. 10.1186/s12889-017-4315-7 PMC5420139 28476140

[44] Keramati MR, Manavifar L, Badiee Z, Sadeghian MH, Farhangi H, Mood MB. Correlation between blood lead concentration and iron deficiency in Iranian children. Nigerian Medical Journal. 2013 Sep 1;54(5):325. 10.4103/0300-1652.122353 PMC3883233 24403711

[45] Słota M, Wąsik M, Stołtny T, Machoń-Grecka A, Kasperczyk A, Bellanti F, Dobrakowski M, Chwalba A, Kasperczyk S. Relationship between lead absorption and iron status and its association with oxidative stress markers in lead-exposed workers. J Trace Elem Med Biol. 2021 Dec;1:68:126841. 10.1016/j.jtemb.2021.126841 34438315

[46] Badawy MI, Wahaab RA, Abou Waly HF. Petroleum and chlorinated hydrocarbons in water from Lake Manzala and associated canals. Bulletin of environmental contamination and toxicology. 1995 Aug 1;55(2). 10.1007/BF00203018 7579932

[47] Abdel-Moati MA, El-Sammak AA. Man-made impact on the geochemistry of the Nile Delta Lakes. A study of metals concentrations in sediments. Water Air Soil Pollut. 1997 Jul;97:413–29.

